# Age-Related Modifications of Diffusion Tensor Imaging Parameters and White Matter Hyperintensities as Inter-Dependent Processes

**DOI:** 10.3389/fnagi.2015.00255

**Published:** 2016-01-19

**Authors:** Amandine Pelletier, Olivier Periot, Bixente Dilharreguy, Bassem Hiba, Martine Bordessoules, Sandra Chanraud, Karine Pérès, Hélène Amieva, Jean-François Dartigues, Michèle Allard, Gwénaëlle Catheline

**Affiliations:** ^1^INCIA, UMR 5287, Université de BordeauxTalence, France; ^2^Centre National de la Recherche Scientifique, INCIA, UMR 5287Talence, France; ^3^ISPED, Centre ISPED, Institut National de la Santé et de la Recherche Médicale U 1219, Université de BordeauxBordeaux, France; ^4^CHU de BordeauxBordeaux, France; ^5^RMSB, UMR 5536Bordeaux, France; ^6^EPHEBordeaux, France

**Keywords:** white matter hyperintensities, diffusion tensor imaging, aging, normal appearing white matter

## Abstract

Microstructural changes of White Matter (WM) associated with aging have been widely described through Diffusion Tensor Imaging (DTI) parameters. In parallel, White Matter Hyperintensities (WMH) as observed on a T2-weighted MRI are extremely common in older individuals. However, few studies have investigated both phenomena conjointly. The present study investigates aging effects on DTI parameters in absence and in presence of WMH. Diffusion maps were constructed based on 21 directions DTI scans of young adults (*n* = 19, mean age = 33 *SD* = 7.4) and two age-matched groups of older adults, one presenting low-level-WMH (*n* = 20, mean age = 78, *SD* = 3.2) and one presenting high-level-WMH (*n* = 20, mean age = 79, *SD* = 5.4). Older subjects with low-level-WMH presented modifications of DTI parameters in comparison to younger subjects, fitting with the DTI pattern classically described in aging, i.e., Fractional Anisotropy (FA) decrease/Radial Diffusivity (RD) increase. Furthermore, older subjects with high-level-WMH showed higher DTI modifications in Normal Appearing White Matter (NAWM) in comparison to those with low-level-WMH. Finally, in older subjects with high-level-WMH, FA, and RD values of NAWM were associated with to WMH burden. Therefore, our findings suggest that DTI modifications and the presence of WMH would be two inter-dependent processes but occurring within different temporal windows. DTI changes would reflect the early phase of white matter changes and WMH would appear as a consequence of those changes.

## Introduction

Aging is associated with widespread brain structural modifications both in gray matter (GM) and in white matter (WM) compartments. The detailed description of WM modifications was made possible using the development of Diffusion Tensor Imaging (DTI). DTI is an established method for studying *in vivo* the WM pathways and has the ability to reveal structural properties of the WM (Basser et al., 2000; Le Bihan et al., 2001; Behrens et al., 2003) by measuring water diffusion at mesoscopic resolution in brain tissue. It has been shown that axonal structure, cell membrane, and myelin sheath strongly influence water diffusion. DTI is sensitive to the magnitude and orientation of water displacement throughout tissue, and such information can be exploited through a tensor model to calculate several diffusion parameters. The commonly used parameters are Mean Diffusivity (MD), Fractional Anisotropy (FA), Axial Diffusivity (AD), and Radial Diffusivity (RD). MD represents a global measure of water diffusion, FA represents the degree of directionality of water diffusivity, AD describes the principal direction of fibers (parallel diffusion) and RD describes the perpendicular diffusion of the principal direction. Therefore, DTI investigation has provided new opportunities to explore aging effects on WM *in vivo* (Moseley, 2002; Salat et al., 2005a,b; Sullivan and Pfefferbaum, 2006; Wozniak and Lim, 2006; Malloy et al., 2007; Lebel et al., 2008, 2012; Pagani et al., 2008; Lee et al., 2009a; Jang et al., 2011; Sala et al., 2012). In studies including older subjects, a consistent pattern of DTI parameters modifications has been described: a decrease of FA and an increase of both MD and RD were observed in the major WM tracts (Head et al., 2004; Pfefferbaum et al., 2005; Salat et al., 2005a,b; Sullivan and Pfefferbaum, 2006). Age-related modifications for AD were less consistent (Bennett et al., 2010; Burzynska et al., 2010). The pattern of FA decrease/RD increase, also observed in pathological conditions such as multiple sclerosis (Roosendaal et al., 2009; Liu et al., 2012), certainly reflects demyelination process, whereas the pattern of FA decrease/AD decrease reflects axonal degeneration as observed in callosotomy condition (Concha et al., 2006).

The aging brain is also characterized by the presence of White Matter Hyperintensities (WMH) observed on T2-weighted sequences. The prevalence and severity of these WM signal abnormalities increase with age (Zimmerman et al., 1986; Breteler et al., 1994; De Leeuw et al., 2001) and more than 90% of older subjects (>60 years old) exhibit WMH in brain magnetic resonance imaging (MRI) scans (De Leeuw et al., 2001). WMH are considered as ischemic lesions and as radiological markers of small-vessel cerebrovascular disease (Jeerakathil et al., 2004; DeCarli et al., 2005). This tissue damage is characterized by demyelination, axonal injury, gliosis, microglia invasion, and amyloid accumulation (Fazekas et al., 1993; Pantoni and Garcia, 1997; Smith et al., 2000; Gouw et al., 2008; Wang et al., 2011).

WMH and DTI parameter modifications have been observed in aging MRI studies, and both are associated with age-related changes. However, the association between these two MRI biomarkers was demonstrated only recently (Vernooij et al., 2008; Zhan et al., 2009; Lee et al., 2009b; Chao et al., 2013; Maillard et al., 2013; Leritz et al., 2014; Maniega et al., 2015). In a longitudinal MRI study of healthy older subjects, Maillard et al. (2013) have shown that baseline FA values were good predictors of the occurrence of WMH at the second MRI follow-up, suggesting a pathophysiological continuum between DTI modifications and the occurrence of WMH. However, another study showed that the etiology of WMH does not fully account for all age-related DTI-modifications (Leritz et al., 2014). More recently, a study has explored the structural characteristics of Normal Appearing White Matter (NAWM) in subjects presenting WMH by measuring various MRI biomarkers, including MD, FA, magnetization transfer ratio, and longitudinal relaxation time (Maniega et al., 2015). This study showed an association between the level of deterioration of the NAWM and the level of WMH, suggesting again a common pathophysiological process. Our study addresses this question through the description of DTI parameters measured in NAWM in two different populations: (1) in subjects presenting low-level-WMH and (2) in subjects with high-level-WMH. With this objective in mind, we investigated the relationships between the two phenomena in aging by comparing diffusion maps of the two above mentioned populations with diffusion maps of healthy young subjects with no WMH. Since FA value is a good predictor of the occurrence of WMH, we hypothesized first that subjects with low-level-WMH would present a pattern of FA decrease and RD/AD increase (classically observed in old subjects), and then that the same pattern would be found in the brain areas outside the lesions in subjects with high-level-WMH. Moreover, we posited that FA decrease and RD/AD increase would be associated with the WMH burden in older subjects with high-level-WMH.

## Materials and methods

The study protocol was approved by the ethics committee of University Hospital (Bordeaux, France). Written informed consent was obtained from all participants.

### Subjects

**Figure 1:**
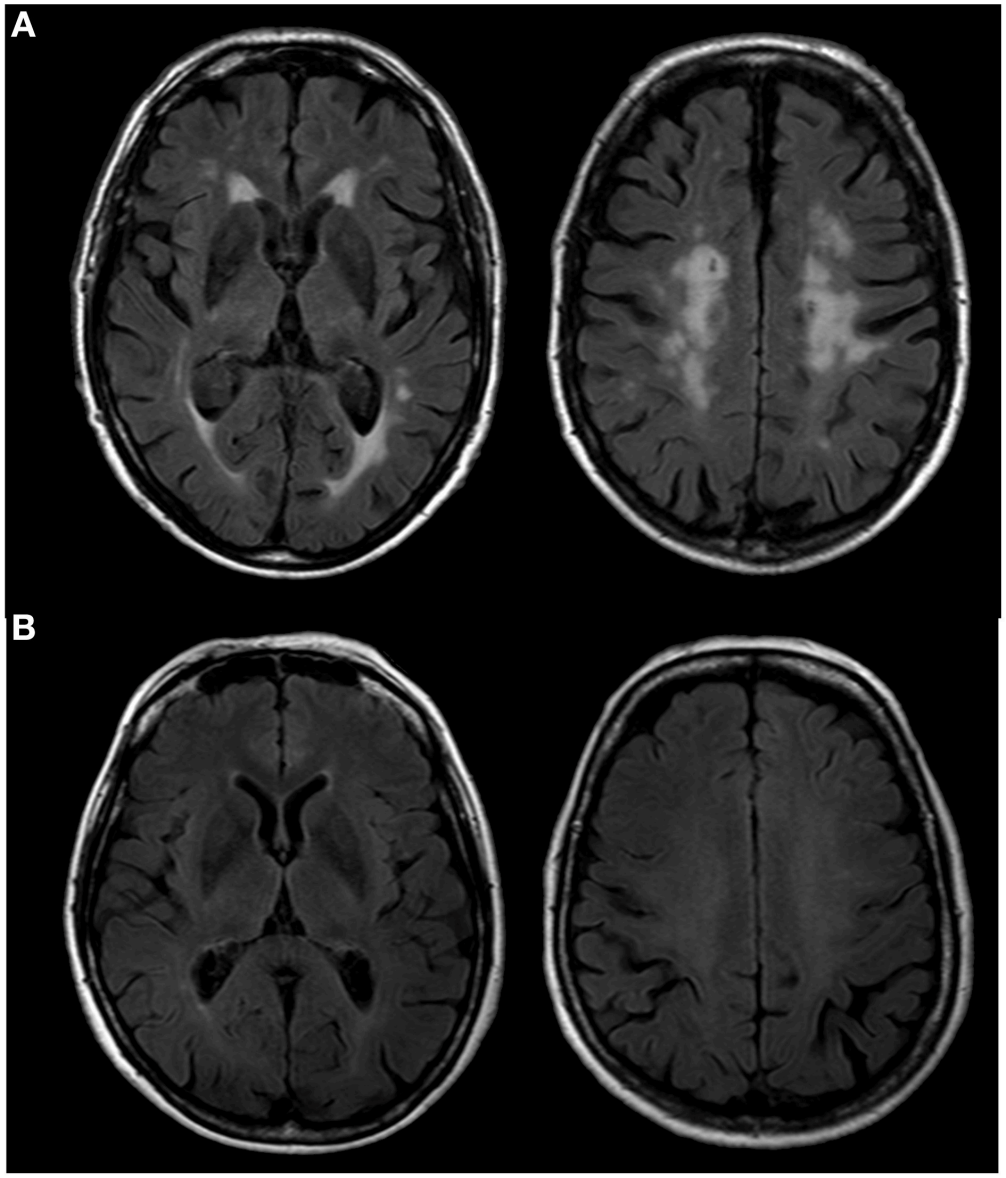
**FLAIR scans of older subjects presenting a high (A) and a low (B) level of WMH according to the Fazekas scale**.

Older subjects were selected from the AMI cohort, an epidemiological study conducted in residents of agricultural communities (Pérès et al., 2012), aged 65 years and older, and who received MRI examinations. Included subjects did not present any acquisition artifacts or cerebral pathologies (e.g., tumor, stroke). They were free of dementia according to DSM-IV criteria and presented normal cognitive functioning as assessed by the Mini Mental State Examination (MMSE; score > 24). Severity of leucoaeriosis was evaluated by two trained operators (AP and MB) on FLAIR scans according to the Fazekas rating scale (Fazekas et al., 1987). Using this scale, we identified 20 subjects with an extensive halo of WMH (grade 3, Figure 1A) [mean age = 79, *SD* = 5.4, (68–86)]. Among subjects with few WMH (grade 1, Figure 1B), we randomly selected 20 subjects [mean age = 78, *SD* = 3.2, (74–85)] matched for age to the group with an extensive halo of WMH. We also included 19 healthy young volunteers [mean age = 33, *SD* = 7.4, (22–47)], for whom the same DTI sequence acquisition was performed. Young subjects did not present any cerebral pathologies or WMH.

### MRI acquisition

MRI scans were obtained using an ACHIEVA 3T scanner (Philips Medical System, Netherlands) with a SENSE 8-channel head coil. For each subject, anatomical, diffusion and Fluid-Attenuated Inversion Recovery (FLAIR) images were obtained.

Anatomical high resolution MRI volumes were acquired in transverse plane for each subject using a 3D MPRAGE T1-weighted sequence with the following parameters: TR = 8.2 ms, TE = 3.5 ms, 7° flip angle, FOV 256 × 256 mm^2^ to cover the whole brain, yielding 180 slices, no gap, voxel size 1 × 1 × 1 mm^3^. Two diffusion-weighted images with opposite polarity, allowing elimination of diffusion imaging gradient cross-terms, were performed using a spin echo single shot EPI sequence with the following parameters: TR = 7646 ms, TE = 60 ms, 90° flip angle, FOV 224 × 224 mm^2^, yielding 60 slices, no gap, voxel size 2 × 2 × 2 mm^3^. One b0 image was acquired and diffusion gradients were applied in 21 non-collinear directions (*b* = 1000 s/mm^2^). To increase signal-to-noise ratio, the sequence was repeated in two successive runs. FLAIR images were also obtained with the following parameters: TR = 11,000 ms, TE = 140 ms, TI = 2800 ms, FOV 230 × 172 mm^2^, yielding 24 slices, gap of 1 mm, voxel size 0.72 × 1.20 × 5 mm^3^. The total scan duration was 38 min. All acquisitions were aligned on the anterior commissure-posterior commissure plane (AC-PC).

### MRI analysis

#### Cerebral volumetric assessment

A Voxel-Based Morphometry (VBM) (Ashburner and Friston, 2000; Good et al., 2001) procedure was used to compute Total Intracranial Volume (TIV). Firstly, the MRI images were spatially normalized and then segmented on their intensity distribution and spatial information derived from the ICBM prior probability maps. The unified segmentation model was extended in the VBM5 toolbox (C. Gaser; http://dbm.neuro.uni-jena.de/vbm) by applying a Hidden Markov Random Field model (Ashburner and Friston, 2005). Secondly, we applied a so-called modulation to each cerebral partition image. During this step, the voxel intensity of the segmented images was adjusted for the strength of deformation derived from the nonlinear spatial normalization process (Jacobian determinants). TIV was computed as the sum of the GM, WM, and CSF volumes.

#### Assessment of white matter hyperintensities (WMH) volumes

WMH volumes were automatically assessed using a Lesion Segmentation Tool for SPM (Schmidt et al., 2012). The procedure included three major steps and operated exclusively in the native space of T1-weighted images. For tissue classification, the T1 image was used to generate a partial volume estimate image and a tissue probability map of WM, GM, and CSF. Next, FLAIR images were co-registered to the T1 images and using intensity distribution of FLAIR images, outliers were detected and lesion belief maps were calculated for the three tissue classes. These lesion belief maps were then summed up and the GM lesion belief map (threshold κ = 0.3) was used as initial lesion map (*Linit*). Finally, a lesion growth model that expands the *Linit* was applied to create lesion maps. WMH volumes were extracted and then normalized by intracranial volumes and log transformed to normalize population variance.

#### DTI parameters

DTI images were processed with FMRIB Software Library (FSL 5.0.2., http://www.fmrib.ox.ac.uk/fsl). For each subject, diffusion-weighted images were co-registered to the reference volume b0 with an affine transformation and were corrected for motion and eddy current distortions. Brain Extraction Tool (BET) was applied to eliminate non-brain voxels. DTI-data were then averaged and FA, AD, RD and MD maps were computed by fitting a tensor model to the raw diffusion data using FMRIB's Diffusion Toolbox.

#### TBSS pipeline

Tract-Based Spatial Statistics (TBSS) pipeline was performed using the standard procedure (Smith et al., 2006). Nonlinear transformations were applied to register individual FA images on the FMRIB58-FA standard template. A mean FA was generated using all the registered individual FA maps. The resulting mean FA image was subsequently thinned (threshold FA value 0.2) to create the mean FA skeleton. Finally, each subject's FA map was projected on the skeleton by searching for maximum FA values perpendicular to the skeleton. The same transformations were applied to the diffusivity maps, i.e., AD, RD, and MD.

#### Extraction of diffusion values outside the lesions in the native space of T1-weighted images

Diffusion maps were co-registered to the T1-weighted images (ANTS software, linear rigid registration algorithm, http://sourceforge.net/projects/advants/). To extract diffusion values on WM outside the area of lesions, two masks were applied on co-registered diffusion maps: the lesion mask obtained from LST procedure and a mask of WM thresholded for *FA* > 0.2. WM outside the WMH area corresponds to the NAWM.

### Statistical analyses

#### Sample characteristics

Mann-Whitney and Pearson Chi-square tests were performed to determine the between-group differences in demographic variables (age, gender), level of education, MMSE scores, WM fractions (WM volumes normalized by TIV), and Mean Arterial Blood Pressure [MABP = diastolic+1/3(systolic-diastolic)].

#### Statistical analyses on whole-brain DTI parameters

The diffusion maps (i.e., FA, MD, AD, and RD maps) used here result from the post-treatment detailed in Section TBSS Pipeline. Firstly, we compared diffusion maps of younger subjects to diffusion maps of older subjects presenting low-level-WMH or high-level-WMH. Secondly, we compared diffusion maps of older subjects with high-level-WMH to those of older subjects with low-level-WMH. Finally, the amounts of WMH burden (WMH volumes normalized by TIV and log transformed) were regressed on diffusion maps in a model adjusted for age and gender for the group with high-level-WMH. For all TBSS analyses, permutations-based statistics with 5000 permutations and threshold-free cluster enhancement (TFCE) (Nichols and Holmes, 2002) were used with a threshold of *p* < 0.001 corrected for multiple comparisons (Smith and Nichols, 2009). The Johns Hopkins University white matter atlas (implemented in FSL package) (Mori et al., 2008) was used to labelize WM tracts.

#### Statistical analyses on DTI parameters outside the WMH area

The diffusion maps (i.e., FA, MD, AD, and RD maps) used here result from the post-treatment detailed in Section Extraction of Diffusion Values Outside the Lesions in the Native Space of T1-Weighted Images. Using Mann and Whitney tests, diffusion parameters of the NAWM of subjects with high-level-WMH were compared to diffusion parameters of the NAWM of subjects with low-level-WMH. Secondly, using Pearson correlations, we tested the relationship between the amount of WMH burden and diffusion parameters outside lesions. These analyses were performed with SPSS package (18.0.0, SPSS Inc.) and a *p* < 0.05 was considered statistically significant.

WMH are classically located in brain regions presenting high FA values (or low diffusion values). The difference in NAWM between the two groups can then be due to the loss of voxels presenting higher FA values (or lower diffusivity values) in the high-level-WMH group. We reran the analysis in common NAWM of the two groups in order to discard this bias (Supplementary Data 1).

## Results

### Demographic analysis

Description of demographic data, MMSE scores and WMH volumes are summarized in Table 1. The mean age of young subjects was 33 years [*SD* = 7.4, (22–47)]. The mean age of older subjects was 78 [*SD* = 3.2, (74–85)] and 79 [*SD* = 5.9, (68–86)] for low-level-WMH and high-level-WMH, respectively. The sex ratio was similar for the three groups. The two groups of older subjects were not statistically different for age, gender, level of education and MMSE scores, and presented the same level of WM atrophy. As expected, the group with high-level-WMH presented a trend for hypertension when compared to individuals with low-level-WMH.

**Table 1:** **Sample characteristics of study participants**.

**Groups**	**Young subjects (*n* = 19)**	**Elderly with low-level-WMH (*n* = 20)**	**Elderly with high-level-WMH (*n* = 20)**	p^*^
Age: Mean (SD)	33 (7.4)	78 (3.2)	79 (5.4)	0.445
Female sex: No.	12	11	7	0.204
Level of education^†^, No. (%)	NA			0.204
1		10	5	
2		8	10	
3		2	5	
MMSE score: Mean (SD)	NA	27.6 (1.2)	27.9 (1.3)	0.134
White matter hyperintensities (% intracranial volume): Mean (SD)	NA	0.13 (0.17)	2.51 (1.46)	<0.001
White matter (% intracranial volume): Mean (SD)	NA	36 (2)	36 (2)	0.968
Mean arterial blood pressure (mm Hg): Mean (SD)	NA	100 (8)	105 (15)	0.069
Periventricular hyperintensity (grade): No.	NA			
0		0	0	
1		20	0	
2		0	4	
3		0	16	
Deep white matter hyperintensity (grade): No.	NA			
0		2	0	
1		18	0	
2		0	11	
3		0	9	

† *1, primary school without diploma*.

*2, primary school validated with diploma*.

*3, short secondary school, long secondary school and university level*.

*NA, not applicable*.

p

* *, p-value for the comparison between the two older groups using Mann and Whitney and Pearson Chi-square tests*.

### Age-related effects on whole-brain DTI parameters

**Figure 2:**
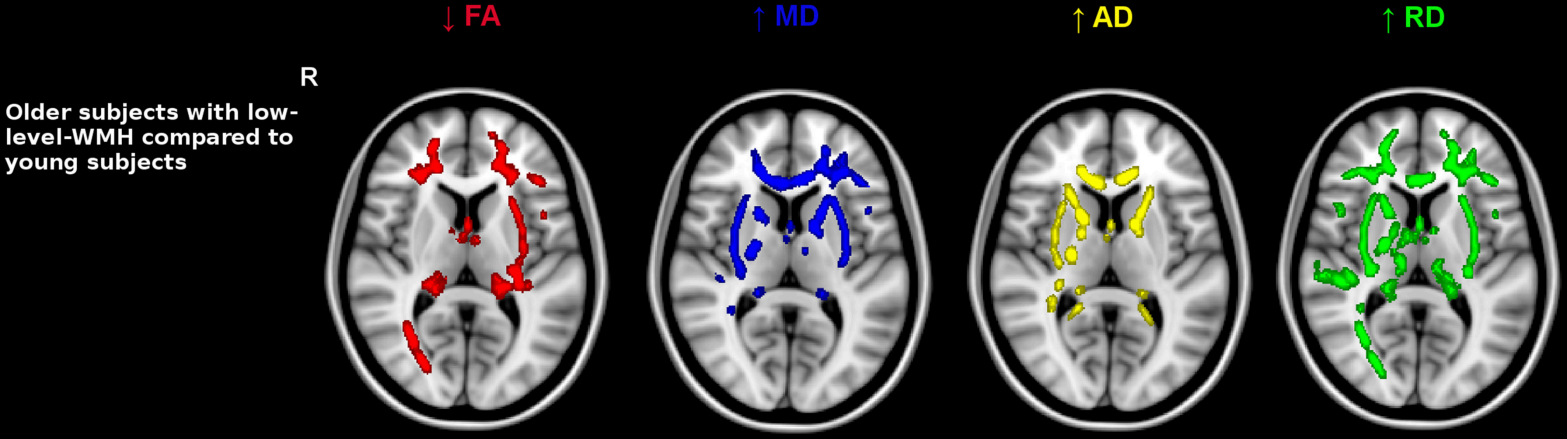
**Comparison between older subjects with low-level-WMH and young subjects**. Results show that the older group with low-level-WMH presented lower FA and higher diffusivity values compared to the younger group. TBSS results displayed at *p* < 0.001, TFCE corrected and overlaid on the MNI template. R, right side.

**Figure 3:**
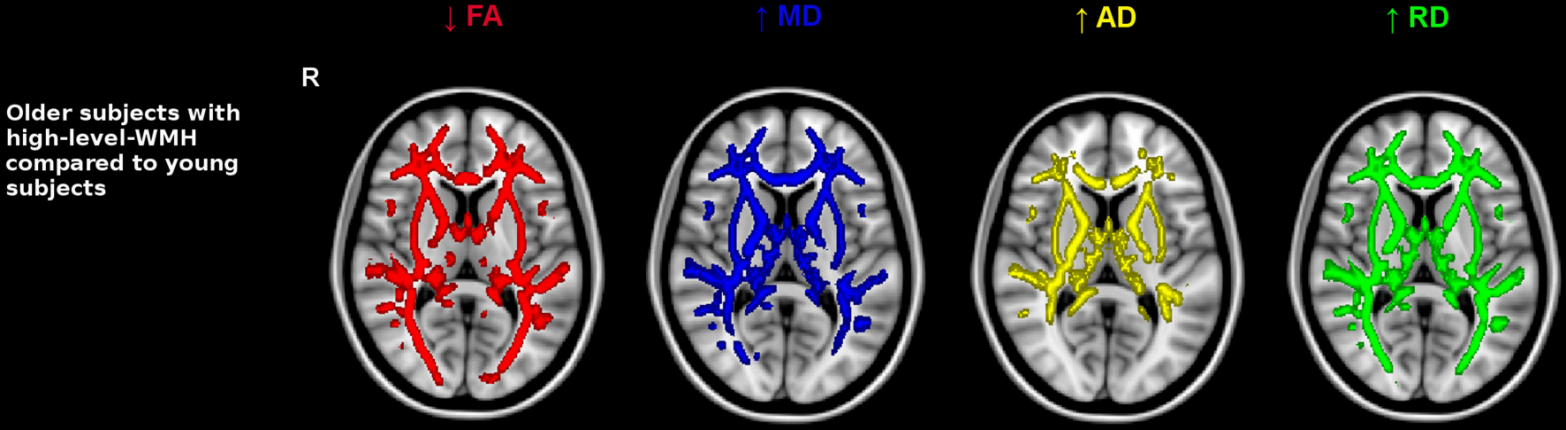
**Comparison between older subjects with high-level-WMH and young subjects**. Results show that the older group with high-level-WMH presented lower FA and higher diffusivity values compared to the younger group. TBSS results displayed at *p* < 0.001, TFCE corrected and overlaid on the MNI template. R, right side.

Older subjects with low-level-WMH presented lower FA values and higher values of all diffusivity parameters compared to younger subjects in various WM regions including the superior corona radiata, genu and body of the corpus callosum, internal and external capsule and the fornix bundle (5000 permutations, TFCE corrected, *p* < 0.001) (Figure 2). As in the previous analysis conducted in old subjects with low-level-WMH, most of the WM regions in old subjects with high-level-WMH presented lower FA values concomitant with higher MD, RD, and AD values when compared to young subjects (5000 permutations, TFCE corrected, *p* < 0.001) (Figure 3). None of the explored WM regions presented lower FA values or higher diffusivity values in the younger group when compared to older subjects with either low or high-level-WMH.

### WMH burden and whole-brain DTI parameters

**Figure 4:**
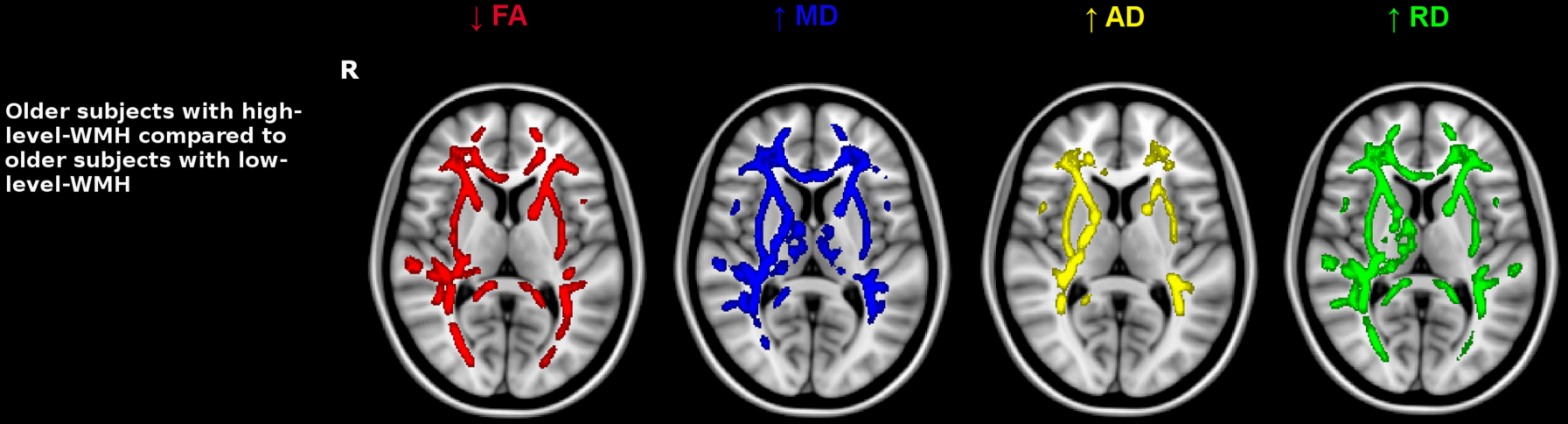
**Impact of WMH burden on diffusion maps**. Older subjects with high-level-WMH were compared to older subjects with low-level-WMH. Results show that older individuals with high-level-WMH presented lower FA values and higher diffusivity values compared to older individuals with low-level-WMH. TBSS results displayed at *p* < 0.001, TFCE corrected and overlaid on the MNI template. R, right side.

**Figure 5:**
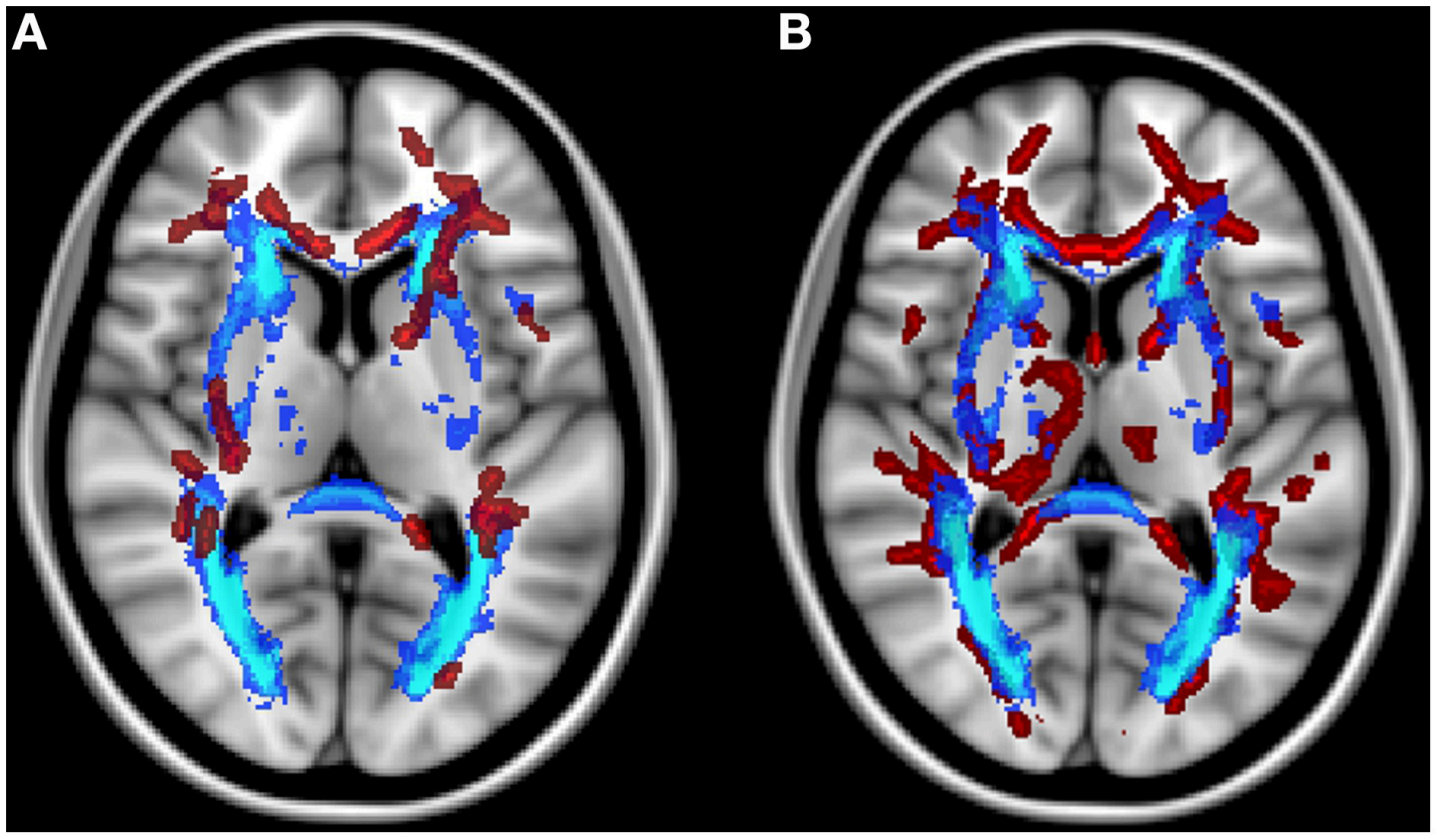
**Modifications of FA values inside and outside the WMH area**. Superimposition of FA voxels (red color) presenting a relationship with WMH burden, and the mean WMH mask (blue color) in older individuals with high-level-WMH. **(A)** Results displayed at *p* < 0.001 demonstrate that FA modifications were mainly located inside the mean WMH mask (blue color) **(B)** Results displayed at *p* < 0.05 demonstrate that FA modifications were also located outside the mean WMH mask (blue color). TBSS results were overlaid on the MNI template.

Older subjects with high-level-WMH presented lower FA values and higher diffusivity values in the whole WM when compared to those with low-level-WMH. Interestingly, these modifications were not only located inside the WMH area but also outside, in the NAWM (5000 permutations, TFCE-corrected, *p* < 0.001) (Figure 4). None of the WM regions investigated in subjects with low-level-WMH presented lower FA values or higher diffusivity values when compared to subjects with high-level-WMH. Moreover, in subjects with high-level-WMH, TBSS analyses revealed a negative relationship between WMH volumes and FA values, and a positive relationship between WMH volumes and all diffusivity values (data not shown) mainly in corpus callosum, anterior limb of internal capsule, anterior corona radiata and thalamic radiations, cerebral peduncles (5000 permutations, TFCE-corrected, *p* < 0.001) (Figure 5A). At a lower statistical threshold (*p* < 0.05), significant clusters extended to the whole white matter (Figure 5B). The superimposition of TBSS results for FA voxels on the mean WMH mask, demonstrated that FA modifications were located inside (Figure 5A) and also outside the mean WMH area (Figure 5B). No positive relationship was observed between WMH volumes and FA values, and no negative relationship was observed between WMH volumes and diffusivity values.

### WMH burden and DTI parameters outside the WMH area

**Figure 6:**
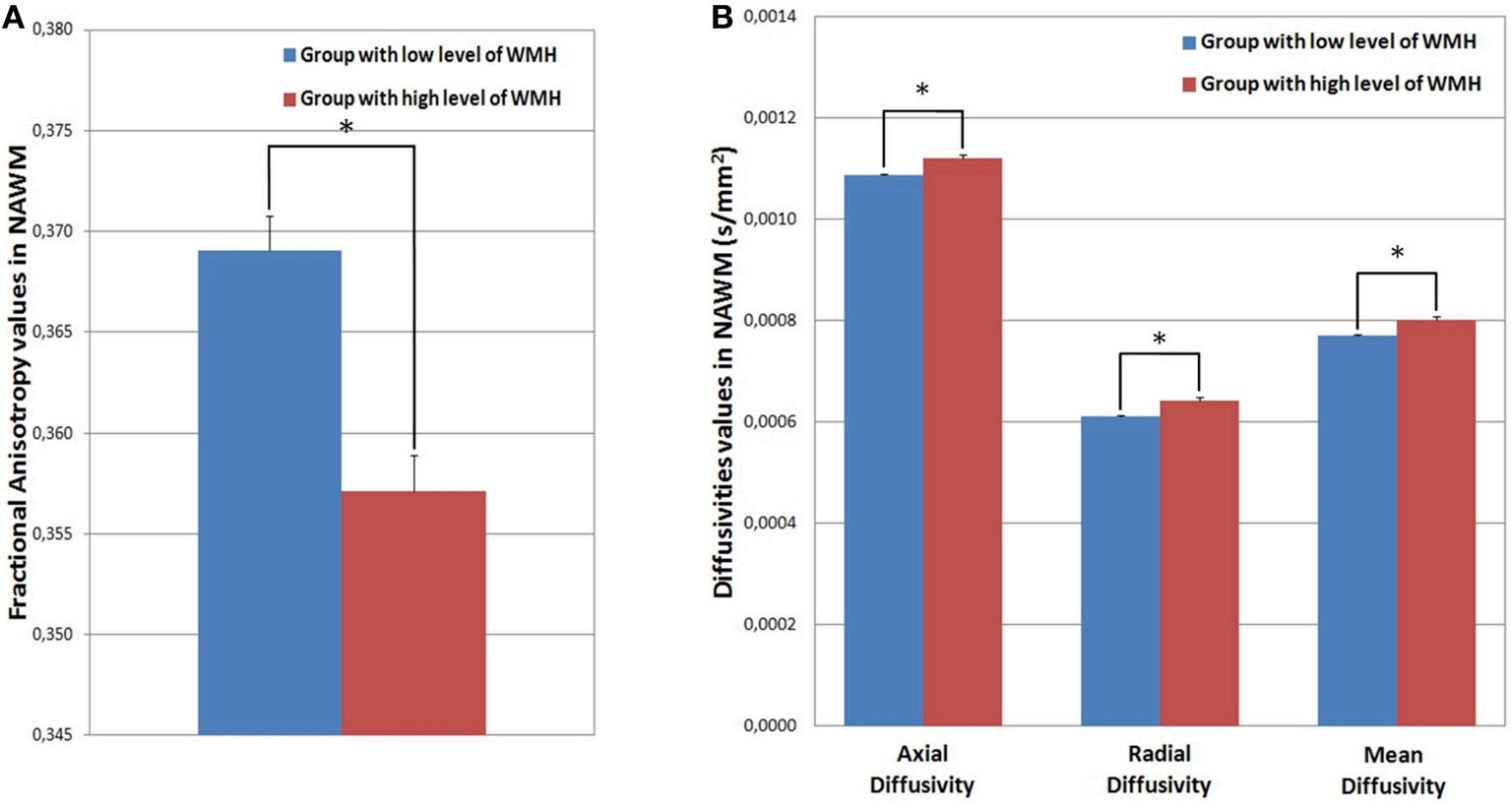
**(A)** Mean FA values extracted in NAWM by group; **(B)** Mean AD, RD, and MD values extracted in NAWM by group. The NAWM of the group with high-level-WMH presented significantly lower FA (Mann and Whitney, *p* < 0.001) and higher AD (Mann and Whitney, *p* < 0.001), RD (Mann and Whitney, *p* < 0.001), and MD (Mann and Whitney, *p* < 0.001) values compared to the NAWM of the group with low-level-WMH. ^*^p < 0.001.

**Figure 7:**
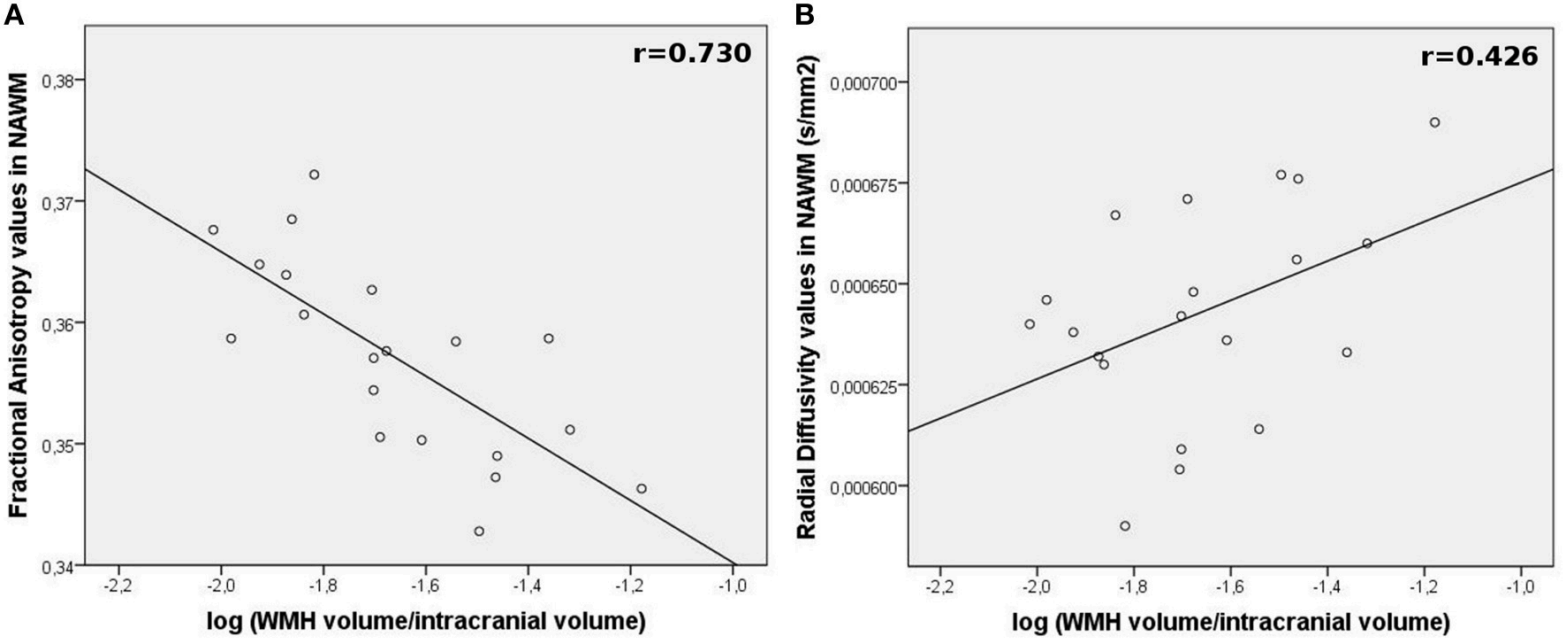
**Relationship between WMH burden and DTI parameters modifications in the NAWM (outside lesions) in high-level-WMH group**. Correlation plots between **(A)** WMH volumes and mean FA values or **(B)** mean RD values of the NAWM for each subject.

The NAWM of the high-level-WMH group presented significantly lower FA values (Mann and Whitney test, *p* < 0.001) (Figure 6A) as well as higher MD (Mann and Whitney test, *p* < 0.001), AD (Mann and Whitney test, *p* < 0.001), and RD (Mann and Whitney test, *p* < 0.001) values compared to the NAWM of the low-level-WMH group (Figure 6B). This analysis was reran in common NAWM to both groups, and similar results were obtained (Supplementary Data). For the high-level-WMH group, a significant correlation was found between the amount of WMH burden and FA values (*r* = −0.731, *p* < 0.001, Figure 7A) and RD values (*r* = 0.428, *p* = 0.030, Figure 7B) measured on the NAWM. A trend for significance was observed for MD (*r* = 0.338, *p* = 0.073) but no effects were observed for AD (*r* = 0.195, *p* = 0.205).

## Discussion

In this study, we observed that even in absence of WMH, older subjects presented WM diffusion changes compared to younger subjects; these modifications follow the same DTI pattern as the one observed in subjects with high-level-WMH. Furthermore, alterations of microstructural properties of the NAWM were greater in older subjects with high-level-WMH compared to those with low-level-WMH. Finally, relationships between WMH burden and diffusion values of the NAWM were found in older subjects with high-level-WMH.

The modifications of DTI parameters in the brain of old subjects were characterized by lower FA and higher diffusivity values (i.e., MD, AD, and RD) in widespread regions, suggesting age-related microstructural alterations of high amplitude since these findings were detectable in a small sample size. Moreover, the pattern revealed here fits perfectly with the well described age-related changes of DTI parameters, i.e., decreased FA values and increased MD, RD, and AD values. In our study, older subjects with low-level-WMH presented DTI parameter modifications mainly in the anterior part of the brain, which is in accordance with the antero-posterior gradient observed with age (Bartzokis, 2004; Head et al., 2004; Pfefferbaum et al., 2005; Salat et al., 2005b; Yoon et al., 2008). In accordance with our results, a recent study performed in a large sample of 126 non-demented individuals (43–85 years old) described a specific age effect in anterior part of the brain after adjustment by the WMH volumes suggesting that the WMH burden does not fully account for all age-related microstructural WM damages (Leritz et al., 2014).

Moreover, as previously described (Bennett et al., 2010; Burzynska et al., 2010), a spatial overlap was observed in the present study between FA and RD variations. Interestingly, we observed that RD modifications were more extended to the posterior part of the brain, suggesting that the RD parameter could be more sensitive to age-related changes. If some discrepancies exist in the literature concerning age-related effects on AD, RD is always described as a measure that increases with age and as the most sensitive to age (Vernooij et al., 2008; Davis et al., 2009; Zahr et al., 2009; Bennett et al., 2010; Sullivan et al., 2010; Sala et al., 2012). More precisely, the genu of the corpus callosum and the association tracts connecting anterior to posterior regions (e.g., superior longitudinal fasciculus) consistently exhibit age-related increases in RD values (Bennett et al., 2010; Burzynska et al., 2010). Finally, our findings suggest that age-related changes of FA are more likely driven by RD changes, classically associated with myelin breakdown (Bartzokis, 2004). It should be noted that RD increase could also reflect changes in the WM compartment other than myelin breakdown, such as reduced neuroglia concentration and changes associated with gliosis (Beaulieu and Allen, 1994). Conclusions about biophysical substrates from DTI metrics should be drawn with caution (Wheeler-Kingshott and Cercignani, 2009).

The comparison between the two groups of old subjects revealed that DTI alterations were identified not only in the regions holding WMH (i.e., periventricular and deep cortical regions) but also in peripheral regions. Indeed, older subjects with high-level-WMH presented lower FA values and higher values of all diffusivity parameters (i.e., AD, RD, and MD) compared to subjects with low-level-WMH, not only in anterior brain regions but also in the whole WM, thereby suggesting that the presence of WMH was associated with an amplification of age-related microstructural alterations. Neither age nor WM atrophy can explain this difference since the two groups are not different for those parameters. Diet habits (Pelletier et al., 2015) and/or physical activity practice (Tseng et al., 2013) could explain the differences observed between the two older groups. The present study also indicated a correlation between FA or RD values of the remaining NAWM and WMH volumes in the older group with high-level-WMH. This association has also been observed in recent studies based on larger sample sizes (de Groot et al., 2013; Leritz et al., 2014; Maniega et al., 2015). Although cross sectional design does not allow to conclude on a causal relationship, it could be hypothesized that DTI abnormalities are part of a continuum of WM degeneration with WMH being the ultimate phase. In this way, DTI would be more sensitive at an early phase for detecting age-related WM alteration. In accordance with this hypothesis, a recent longitudinal study performed on a large sample of old subjects has demonstrated a risk of incident WMH when baseline FA value is low (Maillard et al., 2013).

Regarding methodological considerations, our study presents some limitations. The problem of misalignments due to ventricular enlargement in old subjects is an important issue. We used here the default template, constructed from young subjects. However, we reran the analysis using the ENIGMA template, which could is more representative of our population because it was created from 400 healthy individuals aged 18–85 (Jahanshad et al., 2013), and same results were observed. Moreover, despite the widespread use of TBSS, the skeleton method is questionable (Jones and Cercignani, 2010; Edden and Jones, 2011; Zalesky, 2011; Schwarz et al., 2014). Indeed, subtle local differences in peripheral regions cannot be detected with the skeleton. Moreover, regarding the skeleton projection, it has been shown that the alignment is reasonable between voxels exhibiting common FA values, but was not necessarily between anatomical concordant voxels (Zalesky, 2011).

To conclude, the present study reveals that age-related DTI modifications can be detectable in a small sample size of old subjects presenting no WMH. Moreover, our findings suggest that microstructural alterations and presence of WMH are two inter-dependent phenomena occurring through on the same continuum, with DTI modifications being the earliest phenomenon followed by WMH. These findings should be taken into account for the interpretation of DTI changes in aging subjects. Indeed, diffusion changes are currently interpreted as the result of a neurodegenerative process, occurring after gray matter alterations but according to the continuum theory it would rather reflect cardiovascular risks (Maillard et al., 2012; Salat et al., 2012). Moreover, if future longitudinal analyses on WM tissue confirm that DTI parameter modifications precede the occurrence of WMH, then the use of these DTI parameters as early biomarkers of age-related functional decline should be considered.

## Disclosure

HA has received funding for travel and/or speaker honoraria from Bristol-Myers Squibb, Eisai Inc., Pfizer Inc., UCB, Novartis, and GlaxoSmithKline; and has received research support from Fondation pour la Recherche M9dicale. JD serves on a scientific advisory board for and has received funding for travel from Jansen; has received a gift worth more than US $ 500 from Novartis; holds corporate appointment with Merck Serono; and has received research support from Novartis and Ipsen. MA serves on a scientific advisory board for Novartis neuroimaging; has received research support from Pfizer Inc., IBA CisBio Inc., and GE Healthcare. The remaining authors report no disclosures.

### Conflict of interest statement

The authors declare that the research was conducted in the absence of any commercial or financial relationships that could be construed as a potential conflict of interest.
