# Supplementary material for: Age-Related Modifications of Diffusion Tensor Imaging Parameters and White Matter Hyperintensities as Inter-Dependent Processes
Source: Front Aging Neurosci. 2016 Jan 19;7:255. doi: 10.3389/fnagi.2015.00255 (PMC4718031; doi:10.3389/fnagi.2015.00255)
Supplement: Supplementary file 1 [file DataSheet1.PDF]

Supplementary Data 1:

WMH are classically located in brain regions presenting high FA values (or low diffusivity values). The difference in NAWM between the two groups can then be due to the loss of voxels presenting higher FA values (or lower diffusivity values) in the high-level-WMH group. To discard this bias, we performed a supplementary analysis using only the NAWM regions which are common to old subjects.

Firstly, we reversed the mean WMH mask (MNI space) which allows giving the mean NAWM mask. Secondly, we used the intersection between the mean NAWM mask and the mean FA skeleton mask to extract then the diffusion values from the skeletonized DTI data. This analysis confirms that NAWM of high-level-WMH group present significant lower FA values and higher diffusivity values in comparison to NAWM of the low-level-WMH group. Similarly, a significant correlation is found between the WMH volumes and FA values (r=-0.758, p<0.001) and RD values (r=0.592, p=0.003) of the common NAWM in the high-level-WMH group.

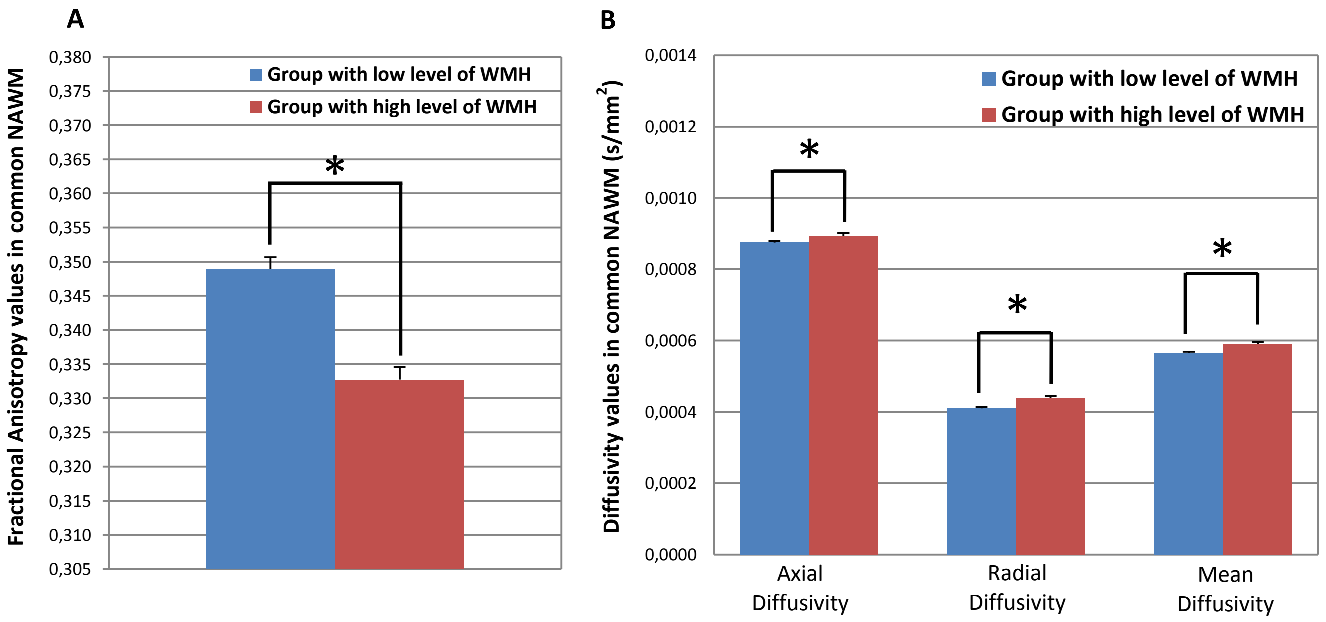

Supplementary Figure 1. Mean FA **(A)** and diffusivity values **(B)** extracted in the common NAWM regions to both groups**.** The NAWM of the group with high-level-WMH presented significantly lower FA (Mann & Whitney, p<0.001) and higher AD (Mann & Whitney, p<0.001), RD (Mann & Whitney, p<0.001) and MD (Mann & Whitney, p<0.001) values compared to the NAWM of the group with low-level-WMH.
